# Dichotomy retreat and aqueous alteration on Noachian Mars recorded in highland remnants

**DOI:** 10.1038/s41561-024-01634-8

**Published:** 2025-01-20

**Authors:** Joseph D. McNeil, Peter Fawdon, Matthew R. Balme, Angela L. Coe, Javier Cuadros, Stuart M. R. Turner

**Affiliations:** 1 School of Physical Sciences, The Open University, Milton Keynes, UK; 2 Natural History Museum, London, UK; 3 School of Environment, Earth and Ecosystem Sciences, The Open University, Milton Keynes, UK; 4 The James Hutton Institute, Aberdeen, UK

**Keywords:** Geomorphology, Mineralogy, Hydrology, Inner planets

## Abstract

The Mawrth Vallis region is a plateau situated on the highland side of Mars’ hemispheric dichotomy boundary. It has a >200-m-thick phyllosilicate-bearing stratigraphic succession that indicates extensive aqueous alteration between 4.1 Ga and 3.7 Ga, during the Noachian Period. In addition, thousands of kilometre-scale isolated mounds in the lowlands north and west of Mawrth Vallis have been identified. Here we use geomorphological and spectroscopic analyses to show that the mounds are erosional remnants that formed through retreat of the highland plateau in the Noachian. Consequently, the escarpment that marks the surface expression of the dichotomy must have receded south-southeast by hundreds of kilometres in this area. Lateral and stratigraphic geochemical variation in the mounds show that widespread, multiphase aqueous alteration occurred in situ across this region in surface and subsurface environments. The mound succession is underlain by a pyroxene-rich unit that represents unaltered material below the regional phyllosilicate-bearing sequence and is unconformably overlain by a thin capping unit that marks the end of large-scale regional aqueous activity. Thus, the mounds contain a stratigraphic record of the onset, evolution and cessation of Noachian aqueous conditions in this region, detailing the environment and climate of Mars at its most habitable.

## Abstract (web summary)

The Martian dichotomy boundary receded hundreds of kilometres in the Mawrth Vallis region and left behind mounds that record changing aqueous conditions during the Noachian (4.1–3.7 Ga), according to a geomorphological and spectroscopic study.

## Main

Lacking plate tectonics and the high erosion rates that recycle Earth’s lithosphere, Mars preserves a comprehensive stratigraphic and geochemical archive of its primordial aqueous environmental conditions^1–4^. Exposures of Noachian-aged (4.1–3.7 billion years ago (Ga)) phyllosilicate-bearing rocks in the highlands surrounding the Mawrth Vallis channel underpin much of our understanding of the role of liquid water on early Mars, which is vital for our knowledge of the evolution of terrestrial planets, planetary habitability and the search for life elsewhere in the Solar System. At Mawrth Vallis, >200 m of Fe/Mg-rich phyllosilicate-bearing strata are overlain by tens of metres of Al-rich phyllosilicate-bearing strata, revealing a complex, multiphase alteration history spanning several hundred million years from the Noachian to early Hesperian^5–11^. The stratigraphic and lateral extent of aqueous crustal alteration is well documented in the highlands surrounding the Mawrth Vallis channel^5,6,10^, but to the north, the highland plateau terminates abruptly at the hemispheric dichotomy boundary.

Thousands of kilometre-scale buttes and mesas (mounds) rise above the lowland plains to the north and west of Mawrth Vallis. These mounds are part of a larger population^12,13^ extending south around the margin of Chryse Planitia, a >4.0 Ga, ~1,200-km-diameter impact basin that straddles the dichotomy boundary^14^. Formed through erosion of regionally extensive ancient material, and associated with antecedent impact craters^12^, the mounds share morphological and thermal characteristics with the phyllosilicate-bearing outcrops of the Mawrth Vallis region. However, the precise stratigraphic relationship between these mounds and the highland plateau has not previously been investigated. Through high-resolution remote sensing analyses of the mounds’ geometry, stratigraphy and geochemistry, we demonstrate that they were once physically contiguous with the phyllosilicate-bearing highlands surrounding Mawrth Vallis. The highland plateau therefore previously extended hundreds of kilometres further north beyond its current position at the edge of Chryse Planitia. The mounds record the migration and evolution of the dichotomy boundary and provide insight into the nature and extent of widespread erosional and alteration processes on early Mars.

## Mound–highland morphostratigraphy and topography

**Fig. 1:**
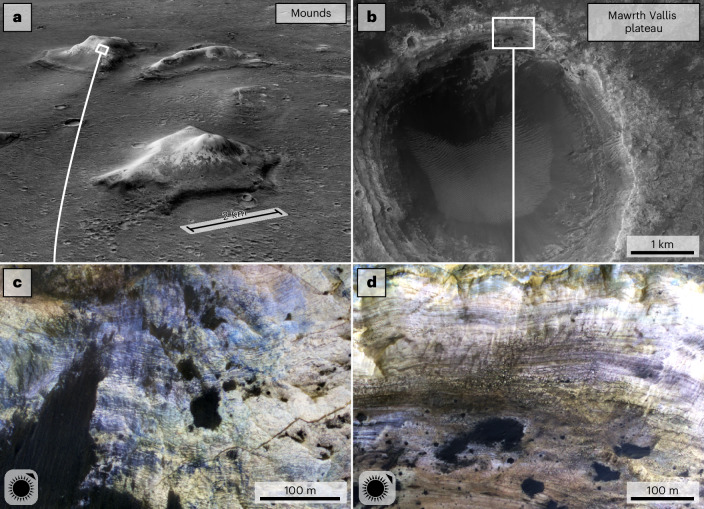
Morphological similarities between the stratigraphy in the mounds and the Mawrth Vallis plateau region. **a**, CTX P21_009102_2063 draped over CTX^64^ DTM (MarsSI^65^) showing three kilometre-scale mounds with bright-toned materials in their upper sections. **b**, HiRISE^66^ RED image ESP_026811_2045 of Tarrafal crater in the highland plateau surrounding Mawrth Vallis. **c**, HiRISE IRB (infrared-red-blue) ESP_063905_2065 showing dark and light-toned horizontal layering in a mound from **a**. **d**, Morphologically similar layering in the northern wall of Tarrafal crater (ESP_026811_2045). Locations of **a** and **b** are shown in Fig. 2a. Illumination angles are shown in the bottom left of **c** and **d**.

The Mawrth Vallis plateau is typified by exposures of bright-toned, horizontally layered, fractured, phyllosilicate-bearing material exposed over >200 m of stratigraphic thickness^5,6,9–11,15^. Morphologically similar exposures are widely detected in the mounds (Fig. 1).

**Fig. 2:**
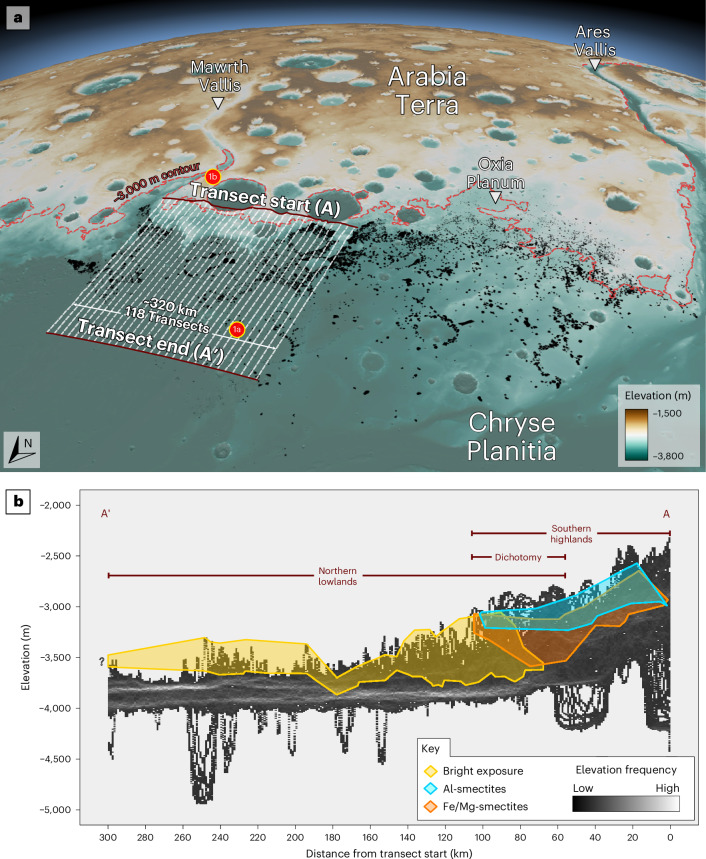
Three-dimensional view of study area with stacked elevation profiles. **a**, A perspective view of the Arabia–Chryse dichotomy region from merged HRSC^67^/MOLA^68^ DTM, (10× exaggeration) showing the southern highlands (brown) and northern lowlands (teal). A total of 10,168 mounds^12^ are shown in black. The −3,000 m contour (dashed burgundy) shows the relationship between the mounds and a line of constant elevation. Transect lines (number reduced for clarity) for **b** are shown. **b**, A histogram showing elevations from 118 topographic transects. Along-stack locations and elevation ranges of bright-toned mound exposures are shown in yellow. Elevation ranges of phyllosilicate minerals on the highland plateau are shown in cyan (Al smectites) and orange (Fe/Mg-smectites), graphically demonstrating the clay compositional stratigraphy common to Noachian-aged terrains^5,7,41,69,70^. A normalized version where vertical fault-derived displacements in the lowlands have been removed is shown in Extended Data Fig. 1.

Data from 118 High Resolution Stereo Camera (HRSC) topographical transects stacked perpendicular to the hemispheric dichotomy show that, in both the mounds and plateau, these exposures crop out at similar elevations and possess comparable thicknesses of morphologically similar material (Fig. 2). This geometric parity, along with their shared morphological characteristics, suggest that the mounds and plateau were originally contiguous. The north-northwest (NNW)–south-southeast (SSE) cross-section (Fig. 2) of the combined deposit is broadly sigmoidal, indicating either greater erosion in deeper regions of the basin and/or thicker accumulations of material close to the present-day plateau.

## Lateral variations in Fe/Mg-phyllosilicate geochemistry

**Fig. 3:**
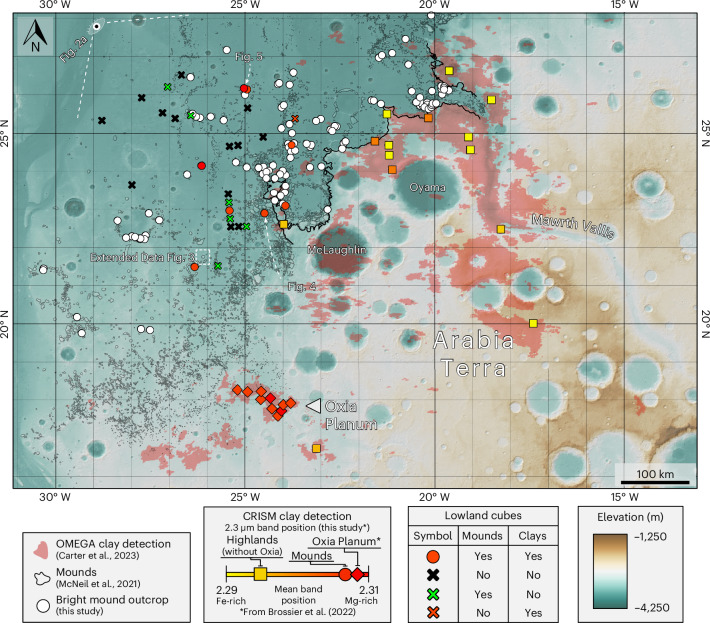
Topography of the study area showing the position of the 2.3 µm metal–OH band in Fe/Mg-rich smectite minerals from CRISM data. Analysis of 28 CRISM cubes in the lowlands (circles and crosses, this study) reveal that smectite spectra from mounds, such as Oxia Planum^22^, exhibit long-shifted 2.3 µm bands (modal per cube), indicating a Mg-rich composition closer to saponite (red circles). The Mawrth Vallis plateau and highlands exhibit short-shifted 2.3 µm bands, signifying a more Fe-rich composition closer to nontronite (yellow-orange squares). Intermediate (orange) values could indicate mixing or intermediate compositions^20^. Dichotomy boundary shown by thick black line. The locations of 109 bright mound outcrops are shown by white points. Detections of phyllosilicate minerals by Observatoire pour la Minéralogie, l’Eau, les Glaces et l’Activité (OMEGA) are shown in pale pink^71^. Basemap: HRSC DTM/hillshade.

The morphologically similar bright-toned exposures on the mounds and highland plateau exhibit absorption features between 1.4 μm and 2.4 µm characteristic of Fe/Mg-smectites^16,17^. Differences in the position of these absorptions indicate variability in the proportion of Fe and Mg present in the smectites^17–20^. Bright mound exposures mostly exhibit long-shifted 2.3 µm bands associated with Mg-smectites (that is, saponite), while the plateau exhibits short-shifted 2.3 µm bands indicative of Fe-smectites (that is, nontronite; Fig. 3). The present-day dichotomy boundary region, situated geographically between these two, exhibits a range of band centres signifying a mixed or intermediate composition. Taken together, these observations indicate a geographical control on Fe/Mg-smectite composition. Similar band centres of phyllosilicates in both the mounds and Oxia Planum^21–23^, the landing site of the ExoMars *Rosalind Franklin* rover^24^, could indicate similar alteration histories; consequently, geochemical investigations by *Rosalind Franklin* may also provide information on regional aqueous processes beyond the landing site.

**Fig. 4:**
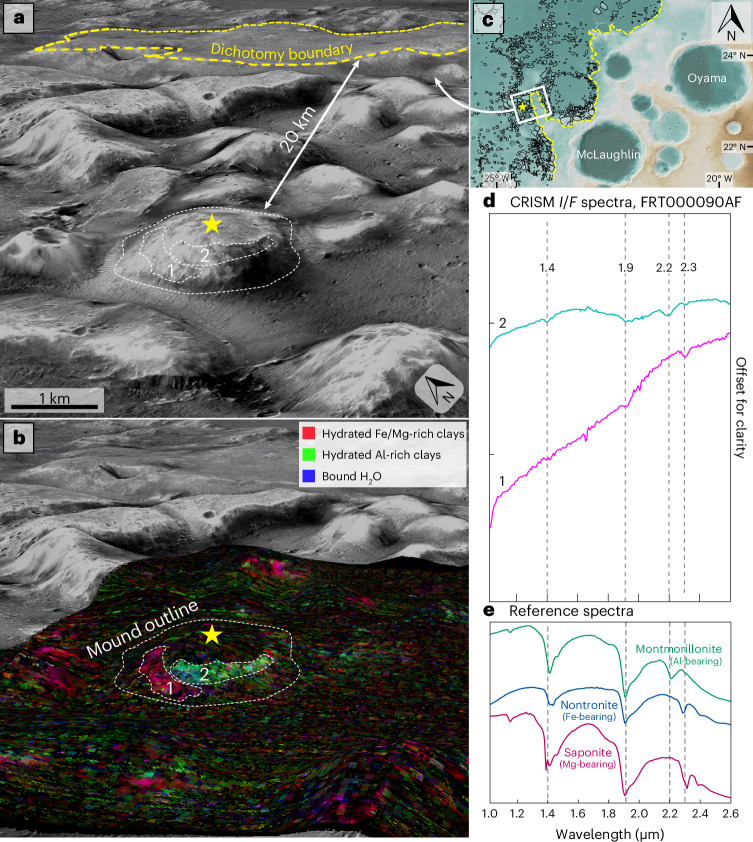
Al-rich phyllosilicate-bearing layer in a dichotomy-proximal mound. **a**, A three-dimensional CTX view of a mound ~20 km from the dichotomy boundary, with its apex indicated by a star. **b**, CRISM^72^ FRT000090AF spectral parameter map (MarsSI) showing Fe/Mg-bearing phyllosilicate-bearing material (region 1, grey dashed lines) and the overlying Al-rich phyllosilicate-bearing material (region 2, grey dashed lines). **c**, Regional context map (HRSC DTM, with dichotomy indicated by the yellow dashed line and mounds in black). **d**, CRISM spectra from (1) and (2). **e**, USGS reference spectra^73^ for montmorillonite (CM20), nontronite (NG-1a) and saponite (SapCa-1).

During the Late Noachian–Early Hesperian, the Mawrth Vallis plateau experienced top-down alteration in pedogenic environments or warm, acidic surface waters, which leached Mg^2+^ cations away from the upper ~50 m of strata, enriching them in insoluble Al^3+^ and Fe^3+^ cations^5–7,10,11^. This montmorillonite/kaolinite-bearing Al unit regularly superposes the preexisting Fe-smectites^7,25^ on the plateau but has been detected in only one dichotomy-proximal mound (Fig. 4). The Al unit here cuts horizontally into the mound and does not drape the topography like in Mawrth Vallis^6^, showing that mound formation continued after the regional Al-unit-forming leaching event.

The lack of Al unit in all but one mound indicates two possible scenarios: (1) the plateau could have either experienced more prolonged alteration than the mounds (as a result of remaining physically connected to the southern highlands, and therefore also the local hydrological system, for longer) or more intense alteration (as a result of better, more interconnected drainage, higher precipitation or more acidic waters); or (2) the Al unit may have initially formed through top-down leaching in the mound region but was subsequently eroded in the later mound-forming event, or is now covered by younger material and not exposed.

## Aqueous history revealed by chemostratigraphic variations

**Fig. 5:**
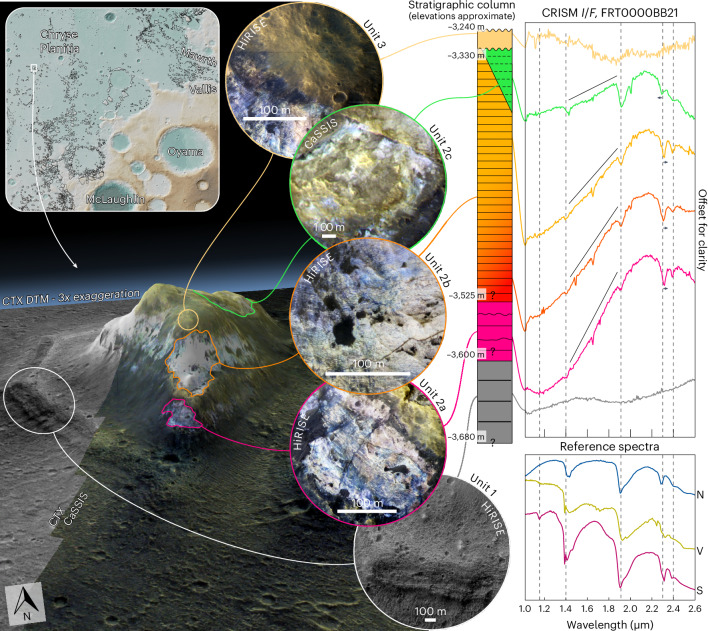
Chemostratigraphy and morphostratigraphy of a well-exposed mound. Three-dimensional Colour and Stereo Surface Imaging System^74^ (CaSSIS) near-infrared/pan/blue (NPB)/CTX DTM image of the mound, with individual morphostratigraphical units 1–3 illustrated in circles using HiRISE IRB and CaSSIS NPB images. To the right is a stratigraphical column derived from these units, with approximate elevations of unit and subunit boundaries indicated. The stratigraphy shows non-hydrated, mafic material at the base (unit 1), overlain by several hundred metres of saponite/ferrosaponite/nontronite-bearing strata (unit 2), in turn unconformably capped by a thin, non-hydrated mafic material (unit 3). The black lines above individual unit 2 spectra indicate an increasing ‘ferrous’ slope with stratigraphic depth. USGS reference spectra^73^ for nontronite (N; Nontronite_NG-1a), vermiculite (V; GDS13_Llano) and saponite (S; SapCa-1) are shown on the bottom right. Mound designated #5647 in the mound shapefile database^12^.

One mound ~250 km from Mawrth Vallis (Fig. 5) exhibits stratigraphically continuous exposure of bright-toned material^12^. We use the high-resolution multi-instrument data that covers this representative mound to exemplify the observable chemostratigraphic and morphostratigraphic variety of the mound–plateau deposit.

The ~80-m-thick basement unit (unit 1) is coarsely layered, yields spectra corresponding to low-Ca pyroxene and is not hydrated (Fig. 5). The overlying unit 2 comprises bright-toned material that is layered at the metre scale, yields spectra corresponding to Fe/Mg-smectites throughout ~350 m of stratigraphic exposure and can be subdivided into three subunits (Fig. 5). The stark difference in stratigraphy, composition and geomorphology between units 1 and 2 indicates a lithological change, due either to different depositional events and/or to post-depositional processes.

Unit 2 exhibits spectra that best correspond to Mg-smectites (for example, saponite) throughout most of its thickness (units 2a/2b*;* Fig. 5). Fe vermiculite is an alternative spectral interpretation (Methods) but is less likely due to spectral shapes between 2.3 μm and 2.5 μm (units 2a/2b). Spectra from the base of unit 2a exhibit a steep slope between 1.0 μm and 1.9 µm, inferred to be related to mixed-valence Fe^2+/3+^-rich clays^8^, indicative either of ferrous components that have avoided alteration at the base of the unit or of additional later alteration in reducing groundwater. The 1.0–1.9 µm spectral slope decreases upsection within unit 2 (Fig. 5), indicating a gradual reduction of ferrous components. Each subunit exhibits High Resolution Imaging Science Experiment (HiRISE)-scale colour variability, suggesting subpixel compositional diversity at Compact Reconnaissance Imaging Spectrometer for Mars (CRISM) scale. Whereas the southwestern side of the mound apex is Mg-smectite rich, the upper northeastern side is rich in Fe-smectites (unit 2c; Fig. 5). Given that strata in this mound are horizontal, this means that the chemostratigraphic Fe-smectite signal must cross-cut the horizontally layered morphostratigraphy; therefore, the Fe-smectite signal was imposed in situ, overprinting preexisting Mg-smectite-bearing strata^26^ at the top of the succession, either during a later subaerial or subaqueous alteration phase^27^ or by coeval lateral variations in alteration environment/parent material. Given the relatively sharp change of mineralogy on either side of the mound, the former is more likely. The Fe-smectite spectra are not indicative of ferrous material (Fig. 5), suggesting later exposure to oxidizing conditions in surface or near-surface environments.

**Extended Data Fig. 2:**
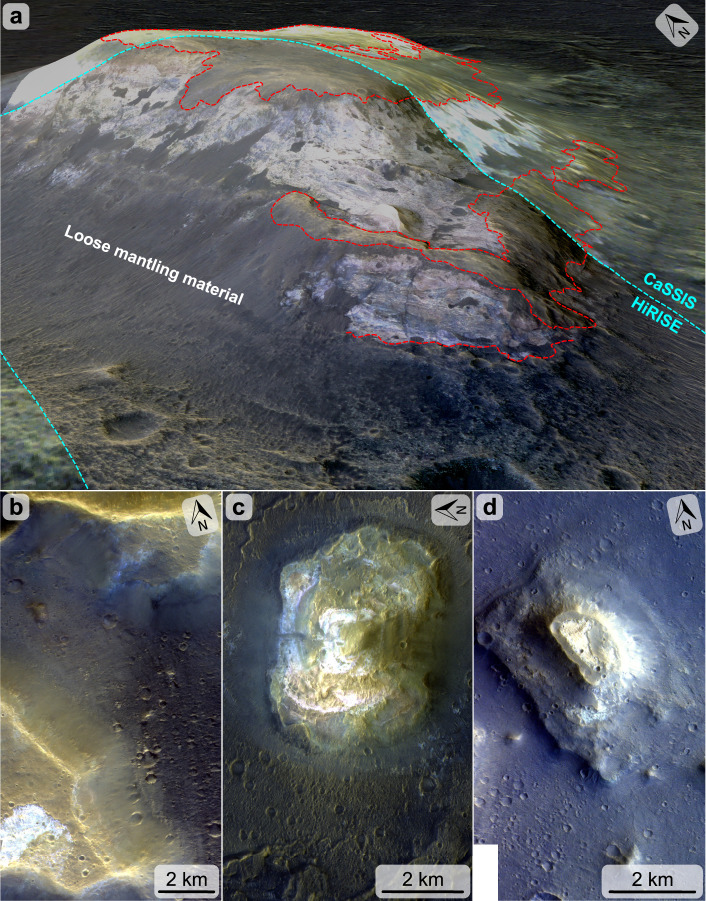
Examples of regional capping material draping mounds across the study area. **a**) HiRISE IRB (IR-RED-BLU) and CaSSIS NPB (NIR-PAN-BLU) draped over HiRISE DTM ( ~ 1 m pixel^−1^), 2× exaggeration. Brown capping unit (edges approximately demarcated by red dashes) drapes the top and southern side of the mound, and must have been deposited after mound erosion. The impact crater cut off at the bottom of the image is ~100 m in diameter, and its ejecta is made of non-hydrated pyroxene-rich material. See supplementary mound information for more detail. **b**) CaSSIS NPB of two mounds at 23.75 W, 24.78 N with flat tops (bottom) and ridged tops showing the capping unit occurring on both. **c**) CaSSIS NPB of a mound at 27.43 W, 19.86 N with the capping unit draping over bright-toned material. **d**) CaSSIS NPB of a mound at 23.90 W, 27.76 N with a flat top and sloping sides dominated by bright-toned material covered in brown capping unit (Unit 3).

**Extended Data Fig. 3:**
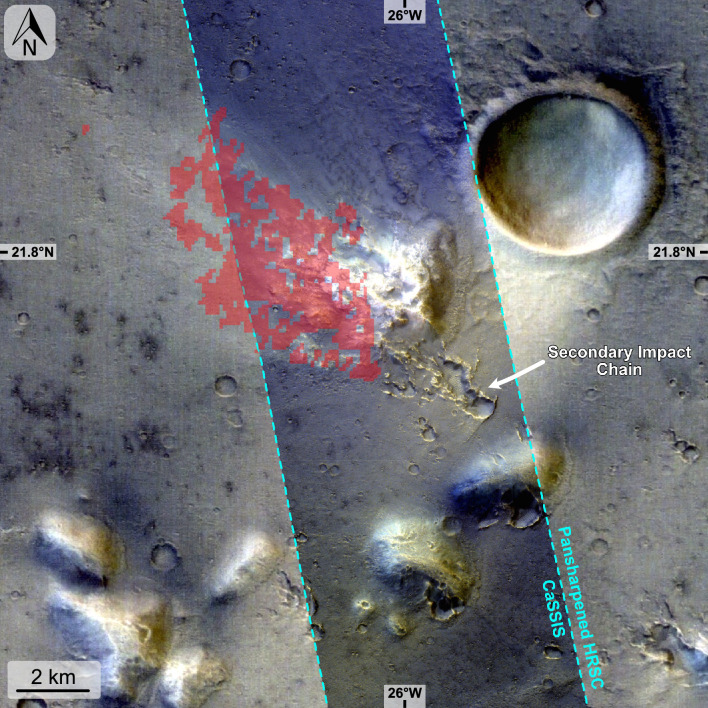
Pansharpened HRSC and NPB CaSSIS showing a mound that has been hit from the southeast by a secondary impactor on its southwestern flank. The source crater from this is likely to be the unnamed ~40 km-diameter impact crater centred at 20.55°N, 24.04°W. The plains to the northwest exhibit bright-toned ejecta oriented from the blast zone, which correspond to detections of Fe/Mg-rich phyllosilicate-bearing material in CRISM survey data (MOCCAS dataset; Carter et al., 2022). Prior to the impact, it is likely that the mound resembled the others around it, and only exhibits this exceptional exposure due to the impact. CaSSIS ID MY37_024829_160_3.

Unit 3, a thin and spectrally bland material, unconformably caps unit 2 (ref. ^12^). Rarely exceeding 10 m in thickness, and regionally extensive (the same material occurs on mounds hundreds of kilometres apart; Extended Data Fig. 2), unit 3 drapes the mound topography^12^. Mounds lacking bright-toned outcrops are covered by unit 3, with bright-toned interiors revealed only where erosion (Extended Data Fig. 2) or secondary impacts (Extended Data Fig. 3) have exposed it. Unit 3 shares characteristics with the ~3.7–3.6 Ga regionally extensive capping unit of the plateau^9^, suggesting a common, widespread emplacement process such as atmospheric dust, distal impact ejecta or volcanic ashfall.

The mound in Fig. 5 is the only known sequence in the circum-Chryse region where hundreds of metres of altered material are bracketed by unaltered material. The stratigraphic succession thus records a transition from non-aqueous subsurface conditions at the bottom (unit 1), to subsurface, reducing, closed aqueous conditions in the basal smectite-bearing materials (unit 2a/2b), to a subaerial or subaqueous, oxidizing aqueous environment in the upper smectite-bearing succession (unit 2c) and, finally, to a thin, non-hydrated capping unit (unit 3). Unit 1, the oldest exposed unit, predates the Mawrth Vallis plateau and is likely to be among the region’s oldest exposed material; it may be stratigraphically equivalent to unexposed, unaltered deposits underlying the clay-bearing plains of Oxia Planum^28^. The boundary between unit 1 and unit 2 marks approximately the onset of aqueous conditions regionally, although the time between the burial of unit 1 and the alteration of unit 2 is unknown. It remains unclear whether unit 1 represents the prealteration protolith for the overlying phyllosilicate-bearing materials of unit 2. The unconformity between unit 2 and unit 3 marks the end of observable regional aqueous alteration.

## Evolution of the dichotomy boundary at Chryse Planitia

Taken together, the similar morphostratigraphy, mineralogy, stratigraphic thicknesses, deposit geometry and geographic settings suggest that during the Noachian the Mawrth Vallis plateau and mounds were contiguous and formed an extensive layered terrain across the highland–lowland crustal transition. Our data show that aqueous alteration occurred in surface and subsurface environments over a much larger region than previously thought, demonstrating that Mars’ climate hosted environments suitable for water–rock interactions over widespread regions during the Noachian. Furthermore, the mounds reveal that the surface expression of the dichotomy boundary—one of Mars’ most fundamental geomorphic features—has been substantially modified in this area with its escarpment relocated hundreds of kilometres further south by subsequent or contemporaneous regional erosion.

**Extended Data Fig. 4:**
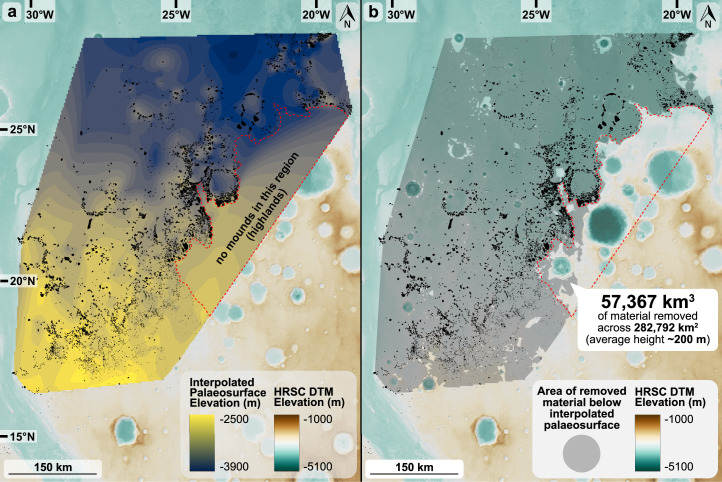
Calculations of removed volumes of material through surface interpolation. **a**) an interpolated surface connecting the upper topographies of all mounds (black) >25 m in height (to avoid sudden unrealistic variations created by adjacent mounds of varying sizes), produced using the natural neighbour tool, and **b**) the same map showing the 282,792 km^2^ region where the present surface lies below the interpolated surface (dark shading); the total volume of removed material was estimated by subtracting the interpolated surface raster from the HRSC MC-11 DTM, using the CutFill tool in ArcGIS Pro.

Lateral scarp retreat has been observed on Mars at both lander^29^ and orbiter^30^ scales, and modification of the dichotomy boundary through Amazonian glaciation^31,32^, Hesperian loading-induced flexure^33,34^ and fretted terrain geomorphic processes^35–37^ is well documented. In comparison with these younger modifications, however, the Noachian dichotomy modification presented here is distinct in both scale and completeness. Along a stretch of 400 km (and possibly up to 2,000 km in Chryse Planitia if the entire mound-forming region is considered), the dichotomy boundary has receded several hundred kilometres. Nearly all intervening material—approximately 57,000 km^3^ over an area of 284,000 km^2^ west of Ares Vallis alone (Extended Data Fig. 4)—has been removed, leaving only remnant mounds. The original extent of the Mawrth Vallis plateau raises the possibility that other regions of the Martian crust may possess similar stratigraphy and mineralogy, but remain unexposed.

The mounds exhibit more trioctahedral Mg-bearing phyllosilicates compared with the plateau’s dioctahedral Fe-bearing phyllosilicates, indicating different alteration histories. Weathering of Fe^2+^-bearing mafic material under subsurface, low-porosity, reducing conditions thermodynamically favours the production of Mg-smectites such as saponite or ferrosaponite^27,38–40^, which is the best match for most mound spectra (see compositional analyses in Supplementary Information). Therefore, the deepest, most distal sections of the deposit, containing more Mg-smectites, must represent alteration in subsurface environments. Fe-smectites, such as nontronite, are observed cross-cutting Mg-smectite-rich strata in mounds (Fig. 5) and also dominate the plateau regions near Mawrth Vallis (Fig. 3). Dioctahedral smectites such as these originate in both subaqueous and subaerial alteration environments on Earth and Mars^8,27^. These lines of evidence suggest that multiple phases of alteration in different aqueous environments affected the mound–highland deposit. In the plateau, prolonged alteration during deposition, whether continuous or intermittent, favoured the formation of dioctahedral Fe-smectites^41,42^, where reduced accommodation space would have led to slow deposition and near-surface aqueous conditions that encouraged alteration contemporaneous with deposition. Material deposited deeper in the basin, where accommodation space was greater, probably accumulated faster, promoting alteration in subsurface diagenetic settings due to continued deposition. This would have resulted in preferential formation and preservation of trioctahedral Mg-smectites that we now see preserved in the mounds.

Such a geographical control on smectite composition may be exacerbated by conversion of Mg-smectite-rich materials into Fe-smectite-rich materials over time, or via more intense weathering in the highland-proximal regions compared to the highland-distal regions. Experimental^19,43,44^ and modelling^26^ work show that Fe-smectites (that is, nontronite)^19,26,43,44^ can form by aqueous alteration of Mg-smectites (that is, saponite) under oxidative and neutral to mildly acidic conditions appropriate to an early Martian, near-surface, aqueous environment^8,27,42^. Furthermore, Mg-smectites dissolve more readily than Fe-smectites across all Mars-realistic pH levels^26,45,46^; therefore, exposure of Fe/Mg-rich smectite-bearing rocks to liquid water for prolonged periods and/or in environments in which water is readily replenished would increase the Fe:Mg ratio of smectites. Lower parts of the stratigraphy and those in highland-distal regions (for example, the mound in Fig. 5) generally did not experience conversion of Mg-smectites to Fe-smectites, as conditions were reducing and water circulation limited, so saponite remained the stable alteration product. By contrast, shallower parts of the deposit, and those closer to the highlands, experienced more oxidizing conditions, with water circulation occurring more readily, and thus any saponite that formed there would have readily converted to nontronite. Oxia Planum—which probably contains vermiculite and/or saponite^21,22,28^—may have been so deeply buried (with limited water–rock interaction and extreme reducing conditions) that it only experienced alteration to vermiculite-facies. This would require less time and/or intensity of weathering than the mounds^47^; as such, not only is Oxia Planum likely to stratigraphically underlie the majority of the Mawrth Vallis plateau sequence^28^, but it may also represent a less altered—and therefore more pristine—section of the Martian crust.

**Extended Data Fig. 5:**
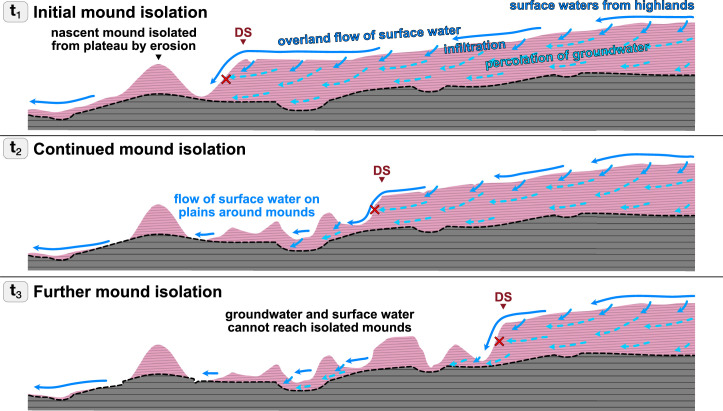
Block diagram of possible landscape evolution showing how surface and groundwaters originating from the highlands are unable to reach newly formed mounds. As time (t_x_) progresses, the dichotomy scarp (DS) backsteps towards the highlands, forming nascent mounds from phyllosilicate-rich mound material (pink). Below the mound material is unaltered mafic material (for example Unit 1 in Fig. 5); separating these is a putative aquiclude. Thus, mounds that belong to the plateau for longer are subject to groundwater and surface water for longer, and therefore will progressively alter to greater degrees.

We hypothesize that highland-distal mounds were separated by erosion from the plateau before highland-proximal mounds, similar to the way that stepwise scarp retreat on Earth is driven by instability and erosion at the escarpment edge^48^. Escarpment retreat probably occurred after alteration, as the absence of a topography-conforming Fe-smectite layer across the mounds indicates that Fe-smectite formation took place in situ within what are now the mound tops before they were eroded into their current form. Lower Mg-smectite-bearing strata show no evidence of conversion to Fe-smectites after their exposure. However, at least some of the mound-forming erosion continued after cessation of regional aqueous activity (probably adjacent to the present dichotomy escarpment), shown by the dichotomy-proximal mound exhibiting an Al unit (Fig. 4). Plateau-proximal mounds left behind as the highlands receded therefore could provide records of the weathering state of the highland boundary at their time of erosion (Extended Data Fig. 5).

**Extended Data Fig. 6:**
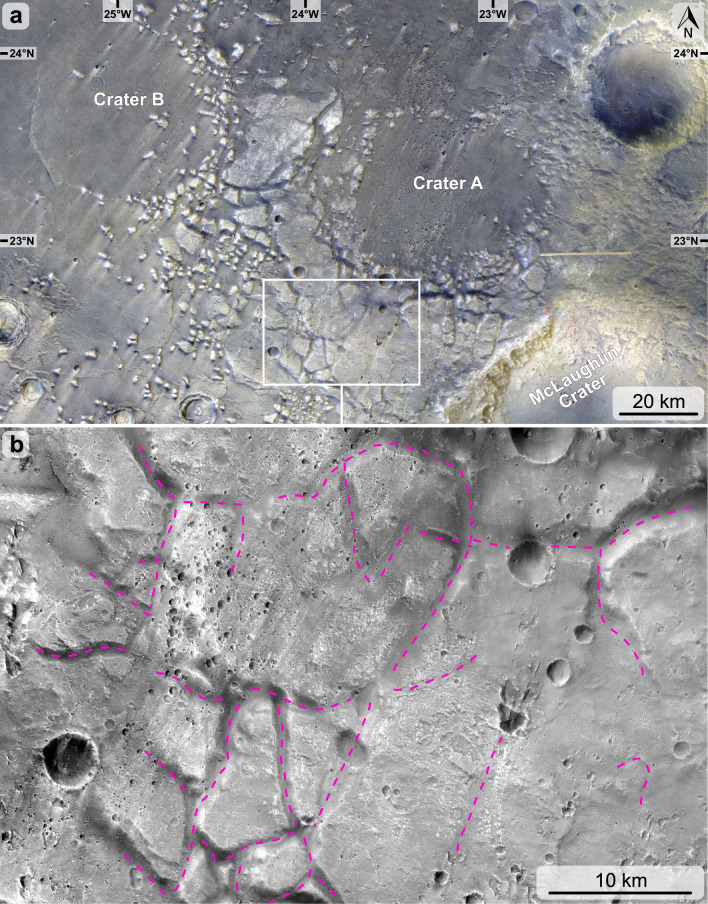
Landscape evolution with possible structural controls. **a**) Pansharpened HRSC showing the degrading plateau west of Mawrth Vallis near McLaughlin Crater. Crater B is likely to be a more eroded version of crater A. The presence of mounds within Crater B shows that the mound material formed after the impact event, and the propensity for mounds near this crater to cluster around its edge shows similarities with other mound populations in the region. **b**) CTX image of linear and curvilinear chasms (pink dashes) at the plateau edge forming mounds; these are likely to be pre-existing fractures that the erosive mechanism(s) that formed the mounds exploited.

The driving processes behind this substantial geomorphic evolution remains unclear. Many of the intermound regions appear to have some degree of structural control (Extended Data Fig. 6), similar to other regions dominated by positive-relief isolated features such as chaos terrains^49–51^ and fretted terrains^35,36^ (for example, Deuteronilus/Protonilus Mensae). It follows that the mounds of Chryse Planitia could have formed through similar mechanisms, such as the sublimation or melting of subsurface ice deposits that exploited preexisting structural weaknesses^35,36^.

## Clues to a northern ocean

The presence of a primordial ocean in Mars’ northern lowlands is contentious^52–56^. Our observations indicate that widespread aqueous alteration affected the mounds and surrounding highlands, suggesting extensive water–rock interactions that could have been facilitated by an early northern ocean.

**Extended Data Fig. 7:**
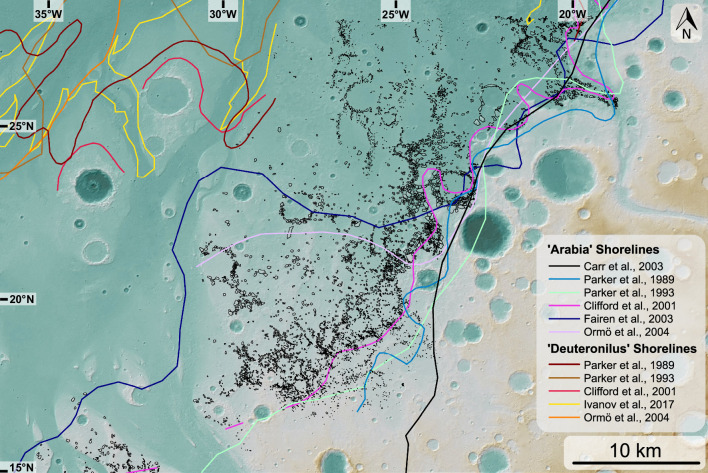
Mounds in Chryse Planitia relative to postulated shorelines. HRSC DTM and hillshade showing highlands (brown) and lowlands (teal), relative to postulated ‘Arabia’ and ‘Deuteronilus’ shorelines. The mounds exist below most of the older ‘Arabia’ shorelines, and above the ‘Deuteronilus’ shoreline.

The mounds mostly lie topographically below the proposed ‘Arabia’ shoreline^55,57–60^ (a Noachian-aged feature) and above the ‘Deuteronilus’ shoreline^55,58,59,61^ (~3.6 Ga (ref. ^61^); Extended Data Fig. 7). A Noachian ocean undergoing forced regression^62^ would have led to subaerial erosion distally from the Arabia shoreline, resulting in a regional-scale unconformity. The mound-forming erosion, which must have occurred between mound–plateau emplacement (4.0–3.9 Ga (ref. ^9^)) and deposition of the unaltered dark plains material (3.7 Ga (ref. ^9^)), fits both temporally and spatially into such a model. The period of substantial erosion and redeposition previously identified in the region at ~3.8 Ga (ref. ^9^) could also be associated with mound erosion. However, Fig. 4 demonstrates that erosion continued into the Early Hesperian, after the formation of the Al unit; therefore, not all of the erosion can be attributed to this 3.8 Ga event, or to the hypothesized forced regression of a Noachian ocean.

The recognition of dramatic landscape evolution at the dichotomy boundary in the Noachian (among other established mechanisms, for example, isostatic rebound or crustal loading from Tharsis^54^) provides an explanation for why proposed ocean shorelines on Mars do not follow equipotential surfaces^55,56^. The proposed Arabia shorelines in the Mawrth Vallis region follow the dichotomy escarpment^55,57–60^ (Extended Data Fig. 7), but our work demonstrates that this feature formed through escarpment retreat and, thus, cannot represent the original expression of the dichotomy or a previous shoreline.

Our analyses of the phyllosilicate-bearing mounds point to a scenario that involves a substantial period of widespread subsurface aqueous alteration, either synchronous with, or through limited overprinting by, surface or near-surface alteration in the plateau-distal areas. This may have been contemporaneous with predominantly surface or near-surface alteration conditions in what is now the plateau-proximal and plateau regions. One reasonable explanation for this wide extent and large stratigraphic thickness of altered material in these locations is a coupled hydrological system and large body of standing water. Phyllosilicate-bearing metre-scale layering, laterally traceable over kilometres within individual exposures (Fig. 5), suggests emplacement in widespread, relatively stable depositional environments compatible with, but not limited to, the presence of a Noachian ocean. Other potential alteration processes such as pedogenesis or a hydrothermal origin may explain parts of the regional alteration but cannot fully explain the entire alteration system that we observe. Pedogenesis explains the alteration of Fe-smectites on the plateau^42,63^ and upper mound stratigraphy but cannot account for the deeper Mg-smectites found in the mound sequence, which formed in the subsurface^27,63^. A hydrothermal origin for the entire alteration sequence appears unlikely owing to the usually localized nature of such activity and the need for a substantial (and here, unrecognized) heat source, although it may explain additional alteration in fractured mounds. Furthermore, the observation of unaltered material at the base of the sequence (Fig. 5) is inconsistent with the alteration process affecting the sequence from the bottom up.

However, regardless of whether the alteration of the mound units was associated with an ancient northern ocean, or other widespread aqueous processes, our work demonstrates a strong connection between the formation and alteration of the mounds, the plateau and the morphology of the dichotomy in this region. The mounds offer an exceptional stratigraphic record of Noachian aqueous processes and landscape evolution along the highland–lowland margin. A future landed mission to the mounds would provide a unique opportunity to investigate dichotomy retreat and assess climatic and environmental changes over time during Mars’ most habitable period.

## Methods

### Bright-toned outcrops

For this study, we used the primary dataset from McNeil et al.^12^, which contains digitized polygons that document the location of mounds in the circum-Chryse region. This dataset is composed of 14,386 individual mound polygons, which demark the geographic locations and record Mars Orbiter Laser Altimeter (MOLA)^68^ Precision Experiment Data Record (PEDR) and HRSC^67^ digital terrain model (DTM) data on mound elevation and height (the elevation difference between the tallest part of each mound and the surrounding plains). For more detail on how these data were acquired, see McNeil et al.^12^.

We constrained our study to all 10,168 mounds in this dataset east of Ares Vallis, as from observational experience, mounds in this region exhibit the best-exposed outcrops of the bright-toned material in which we are interested, although it is unclear whether this is a result of higher post-capping unit erosion, diverse alteration environments or another factor. A total of 158 Context Camera (CTX)^64^ images were used as a basemap, displayed and analysed using Esri ArcGIS Pro software, a geographic information system. An observational survey of all mounds in the study area was conducted, in which each discrete exposure of bright-toned material that existed within the mound polygons at the CTX scale was digitized by hand. These are easily identified as they contrast the darker, dull capping unit that drapes mounds. Where multiple bright-toned exposures occurred in a single mound, these were digitized as separate polygons that retained information about their parent mound polygon through ArcGIS Pro’s zonal statistics tool.

Once all mounds were surveyed for bright-toned exposures, the completed bright-toned exposure polygon database was integrated with topographic data using ArcGIS’s zonal statistics tool to provide the maximum, minimum and average elevations for each exposure. For this, two different datasets were used: the MC-11 HRSC DTM, which covers all surveyed mounds at a resolution of ~50 m per pixel but has irregular artefacts and low-quality regions, and the MOLA PEDR, which details the elevation of points at 300 m intervals along a spacecraft orbital track but does not occur consistently across Mars’ surface and so occurs irregularly within exposure polygons.

### Stacked transects

The isolated nature of the mounds means that a single, linear elevation profile across the highlands and dichotomy and into the lowlands may cross only one or two prominent topographic mounds. To be able to see many mounds in a single transect, the profiles would have to dog-leg multiple times; however, this is suboptimal for observing proximal–distal changes in the mound population relative to the highlands because these changes would be off-axis and inconsistent with the true along-dip variation.

To overcome this, 118 stacked transects were produced that allow all substantially large mounds in a given area to be viewed in a single ‘data cube’, which indicates the propensity for elevations to occur at given distances from the start of the transect set. Using this method has its trade-offs; individual mounds (which are small in comparison with the study area) are unresolvable within the larger data cube, and large topographic features (for example, impact basins, faults and ridges) can skew the data. However, the ability to view the relationship between along-trend distance and topography provides unique insight to the topography of the highland–lowland transitional areas.

We chose a region of the hemispheric dichotomy that was relatively linear (Fig. 2), and drew the initial 318 km transect start line on the highland plateaus both parallel to it and perpendicular to the overall NNW trend of Mawrth Vallis at a bearing of 054°. This meant that the dichotomy escarpment occurred in a more constrained *x*-axis range within the data cube and was more likely to be representative of, and parallel to, any dichotomy margins that previously existed in this region. Using ArcGIS Pro’s ‘Generate Transects Along Lines’ tool, 118 lines 300 km in length were produced perpendicular to the transect start line (Fig. 2), with a spacing of ~2.65 km, which projected NNW across the plateau, down the dichotomy and along the plains, intersecting most large mounds. This number of transects was reached through iteration as a compromise between the ability to capture representative elevations of mounds of all sizes and the resultant size of the data cube. Next, ArcGIS Pro’s ‘Stack Profile’ tool was used across all 118 transects to sample the underlying HRSC DTM every 50 m along strike and produce the stacked elevation dataset. R Studio software was used to visualize the dataset as a histogram, to overlay the locations of bright-toned exposure and Al-rich/Fe/Mg-rich phyllosilicate detection elevation data as vertical lines and points, respectively, at known distances along the transect, and to produce best-fit polygons of these lines to show their spatial ranges in two dimensions as shown in Fig. 2.

### Compositional analyses

To investigate the composition of mounds across the region, we analysed all 28 CRISM^72^ cubes available in the lowland region between Ares Vallis and Mawrth Vallis and 13 CRISM cubes on the highland plateau, the latter focused primarily in the area north of Oyama Crater and west of the Mawrth Vallis channel. CRISM *I*/*F* data (where *I* is the radiance on the sensor and *F* is the expected solar radiance during the observation if there were no atmosphere) were acquired from the National Aeronautics and Space Administration (NASA) Planetary Data System (https://pds.nasa.gov) in full-resolution targeted and half-resolution short formats. Subsequent processing and analyses of the data (following standardized techniques^75,76^) were conducted using the CRISM Analysis Toolkit, a software package developed by the CRISM team as an extension to the Environment for Visualizing Images version 8.8.1 image processing and analysis software. As part of this process, photometric corrections were applied to correct for viewing geometry, and atmospheric corrections using the volcano scan method^77^ were used to distinguish the contribution of the reflected light from the surface from that of the atmosphere.

Cubes were initially surveyed using summary parameters from Viviano-Beck et al.^78^. The D2300 (Fe–OH/Mg–OH 2.3 µm drop-off indicative of Fe/Mg-bearing hydroxylated silicates), BD2210_2 (band depth at Al–OH 2.2 µm absorption indicative of Al-bearing hydroxylated silicates) and BD1900R2 (band depth at 1.9 µm indicative of bound H_2_O) summary products were used to identify phyllosilicate-bearing regions. Other spectral parameter combinations, for example, OLINDEX3 (detects a broad absorption at 1 µm indicative of olivine), LCPINDEX2 (detects a broad absorption at 1.81 µm indicative of low-Ca pyroxene) and HCPINDEX2 (detects a broad absorption at 2.12 µm indicative of high-Ca pyroxene) were used for mafic mineral identification, and BD2355 (band depth at 2.35 µm sensitive to chlorite, prehnite and pumpellyite), D2300 (Fe–OH/Mg–OH 2.3 µm drop-off indicative of Fe/Mg-bearing hydroxylated silicates) and BD2290 (another parameter for the band depth at 2.3 µm, sensitive to Fe/Mg–OH minerals and CO_2_ ice) were used to identify possible changes in Fe/Mg-rich phyllosilicate minerals within a cube. Where available, MarsSI^65^, a webGIS image processing of orbital Mars data in its pipeline, was used to produce additional computed spectral parameters. These compare observed spectra from each pixel with theoretical mineral spectra and produce maps showing the strength of each mineralogical comparison in each pixel for us to identify the best potential locations to extract spectra from in CAT. Spectrally interesting regions of mounds and highlands were ratioed to a corresponding spectrally bland reference region^75,76^. This removed the systematic instrument artefacts and atmospheric residual artefacts, and emphasized the compositional differences between the area of spectral interest and the reference region^11,75,76^.

Spectra were investigated in the 1.0–2.6 µm range. Four key absorptions were required for a positive detection of smectite minerals^8,16,17^: (1) an absorption at 1.4 µm caused by a stretching overtone of OH and of H_2_O, (2) an absorption at 1.9 µm caused by a combination of O–H stretch and bending in H_2_O, (3) an O–H bending and stretching combination in metal–OH groups that produces an absorption at either ~2.2 µm (in the case of Al_2_OH) or 2.3 µm (in the case of Fe_*n*_/Mg_*m*_OH, where 2 ≤ *n* + *m* ≤ 3) and (4) an absorption at 2.4 µm. Lowland CRISM cubes with non-detections of phyllosilicate minerals (due to either a lack of mounds or a lack of exposed bright-toned outcrops on those mounds) are also indicated by crosses in Fig. 3.

For highland cubes, regions that corresponded to Al-rich phyllosilicates and Fe/Mg-rich phyllosilicates (of any kind) were marked with either ‘Fe_Mg’ or ‘Al’ points in ArcGIS Pro. This resulted in several points per highland CRISM cube. Elevations were subsequently tied to these points through the zonal statistics tool, and the distances from each of these points to the regional transect start line were also calculated. These elevations and distances, superposed on the stacked transects in two groups based on Fe/Mg- or Al-rich phyllosilicate content, show the stacked elevation ranges on the highland plateau (Fig. 3).

For exposures of phyllosilicates on mounds, regions of interest that yielded spectra corresponding to Fe/Mg-rich phyllosilicates had their band centres in the windows of interest (that is, 1.4, 1.9, 2.3 and 2.4 µm) manually located and recorded in a database, which was subsequently appended onto the shapefile to merge geographic and compositional information into the same geodatabase. The spectra were compared with United States Geological Survey (USGS) reference spectra^73^ of specific Fe/Mg-rich phyllosilicate phases, and a best-fit composition was determined on the basis of band position and overall spectral shape. In all but one mound, Mg- and Fe-rich smectites were found to be the best fit. Vermiculite, an Fe/Mg-rich clay mineral, was considered owing to its considerable spectral variability but in all but one case was ruled out because of (1) the sharp, relatively symmetrical 2.3 µm absorption, (2) the shape of the spectral shoulder around 2.3 µm and (3) the extremely high maximum intensity between the 2.3 μm and 2.4 µm absorptions^47^. The presence of a minor absorption between 2.22 μm and 2.25 µm throughout the stratigraphic profile could indicate a mixed-layer clay containing both Mg-smectites such as saponite and vermiculite; however, Mg-smectites, particularly saponite, are the better match for the above reasons.

## Online content

Any methods, additional references, Nature Portfolio reporting summaries, source data, extended data, supplementary information, acknowledgements, peer review information; details of author contributions and competing interests; and statements of data and code availability are available at 10.1038/s41561-024-01634-8.

## Supplementary information

Supplementary Information Supplementary Figs. 1–7, Supplementary mound stratigraphy information and Descriptions of supplementary data located at https://doi.org/10.21954/ou.rd.23833443.v1.

## Figures and Tables

**Extended Data Fig. 1:**
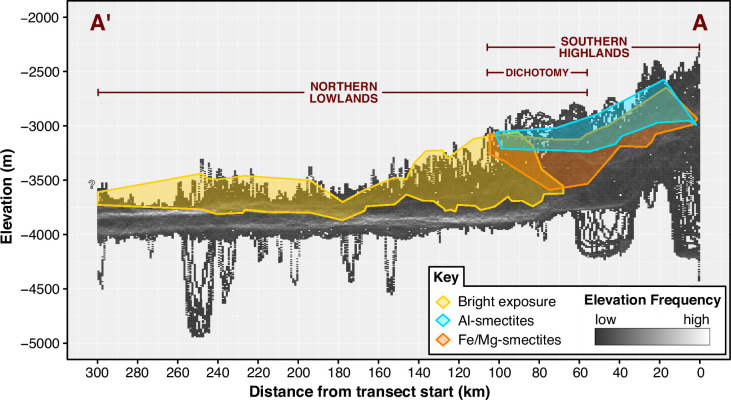
Fault-normalised version of Fig. 2b. The bright mound exposures over 160 km from the transect start have been shifted down by their average upthrown displacement.

## Data Availability

Corresponding data^79^ are available on the Open University’s Open Research Data Online (ORDO) repository at 10.21954/ou.rd.23833443.v1. These data describe (1) the locations, elevations and characteristics of bright-toned exposures on mounds, (2) the position of 1.4, 1.9, 2.3 and 2.4 µm bands of bright-toned exposures that correspond to Fe/Mg-rich and Al-rich phyllosilicates, (3) spectra extracted from regions of interest, (4) the locations and composition of clay detections on the Mawrth Vallis plateau and (5) topographical information corresponding to the transect regions.
