# Supplementary material for: Dichotomy retreat and aqueous alteration on Noachian Mars recorded in highland remnants
Source: Nat Geosci. 2025 Jan 20;18(2):124–32. doi: 10.1038/s41561-024-01634-8 (PMC11810793; doi:10.1038/s41561-024-01634-8)
Supplement: Supplementary file 1 — Supplementary Figs. 1–7, Supplementary mound stratigraphy information and Descriptions of supplementary data located at https://doi.org/10.21954/ou.rd.23833443.v1. [file 41561_2024_1634_MOESM1_ESM.pdf]

nature geoscience

Supplementary information

https://doi.org/10.1038/s41561-024-01634-8

Dichotomy retreat and aqueous alteration on Noachian Mars recorded in highland remnants

In the format provided by the authors and unedited

Supplementary Information for

'Dichotomy retreat and aqueous alteration on Noachian Mars recorded in highland remnants'

J.D. McNeil et al., Nature Geoscience

Table of Contents

Page 2-3: Supplementary Mound Information
Page 4-8: Supplementary Data and Descriptions

Supplementary Mounds Information

Relationship between Unit 1 and 2

The true contact between Unit 1 and Unit 2a in this mound is not directly observable, but key evidence points to Unit 1 being a distinct, older unit that lies stratigraphically below Unit 2.

We do not observe Unit 1 on top of Unit 2 in any location, and where we observe Unit 1, it is the lowest observable section of the stratigraphy. Small, bright-toned outcrops, similar in appearance to Unit 2, appear throughout the basin clearly overlying material that is similar in appearance and stratigraphic position to Unit 1, regardless of elevation.

The >100 m impact crater that has excavated Unit 1 (see Fig. S2a) shows no hydrated material in its ejecta and contains Low-Ca pyroxene, which is typically found in the most ancient terrains on Mars. An impactor of this size is likely to have excavated ~30 m deep, and this is a substantial (~38%) amount of the thickness of Unit 1 so we can be quite sure that there are no clays in Unit 1. This shows that Unit 1 cannot simply be a deeper section of Unit 2 covered by a thin veneer of dark material.

The alternative is that Unit 1 is a younger skirting unit, but Unit 1 is embayed by the Early Hesperian dark plains unit[9] across the basin, so must have been eroded into its current form by the time that unit was emplaced. This would require substantial deposition (~100 m) *and* erosion of Unit 1 in a relatively short space of time, with virtually all other occurrences of Unit 1 removed by the erosion throughout the basin (as we do not observe it in most mounds), which is extremely unlikely. Furthermore, as it is mafic in composition, an igneous source is required. No such volcanic source has been recognised, and emplacement through ashfall/dustfall would not produce the coarse layering observed in this unit, and it would also emplace on the topographic highs of the mound, which we do not observe.

This leaves two possibilities: it underlies the clay-bearing material as a distinct unit, or it is a skirting unit emplaced after mound erosion and cessation of aqueous processes, but before the emplacement of the dark plains material. The fact that it follows the general geometry of the mound it is a part of could suggest either, but if it was emplaced as a post-erosional unit, we might expect to see considerably more of it across the basin, as the primary mound erosion was the most destructive erosive event in the regional history.

Taken at face value, these observations suggest that Unit 1 stratigraphically underlies Unit 2.

Supplementary Data

All supplementary data are available at https://doi.org/10.21954/ou.rd.23833443.v1, and are described below.

Bright_Mound_Outcrops.zip

Shapefiles showing the locations of bright-toned mound outcrops digitised using CTX data.

Field Header	Description
OBJECTID	Numerical designation for each outcrop.
Shape	Polygon (across all individuals).
Outcrop_Perimeter_m	The perimeter of the digitised outcrop in metres.
Outcrop_Area_m^2	The area of the digitised outcrop in square metres.
Elevation_min_m	The value of the pixel with minimum elevation within or partially within the area of the outcrop, taken from the HRSC DTM, in metres.
Elevation_max_m	The value of the pixel with maximum elevation within or partially within the area of the outcrop, taken from the HRSC DTM, in metres.
Elevation_mean_m	The mean elevation value of all pixels within or partially within the area of the outcrop, taken from the HRSC DTM, in metres.
Lon	Longitude of centre point.
Lat	Latitude of centre point.

MawrthPlateau_CRISM_FRT_Detections.zip

Point shapefiles showing the location and composition of points on the Mawrth Vallis plateau corresponding to phyllosilicate mineralogy. The points are recorded as Fe_Mg (Fe/Mg-rich) or Al (Al-rich).

**Field Header**	**Description**
OBJECTID	Numerical designation for each point occurrence.
Composition	Fe_Mg (denoting Fe/Mg-rich phyllosilicates) or Al (denoting Al-rich phyllosilicates).
Near_Dist	Distance, in kilometres, to the transect start line.
CRISM_ID	Product ID of the FRT CRISM cube from which the observations of mineralogy were taken.
Point_X	Longitude
Point_Y	Latitude
Elevation_m	The elevation value of the HRSC DTM pixel in which the point falls, in metres.

Mound_Clay_Detections_Polygons.zip

Polygons showing the location and composition of bright-toned mound outcrops that contain phyllosilicate minerals observed using FRT CRISM hyperspectral data. This is identical to the next dataset, but in polygon form.

Field Header	Description
OBJECTID	Numerical designation for each outcrop.
Shape	Points (across all individuals).
Target_ID	ID of FRT CRISM data from which observation is taken, followed by a numerical designation of an individual spectrum taken from the area of interest this shape covers.
MIN	The value of the pixel with minimum elevation within or partially within the area of the outcrop, taken from the HRSC DTM, in metres.
MAX	The value of the pixel with maximum elevation within or partially within the area of the outcrop, taken from the HRSC DTM, in metres.
RANGE	The MAX subtract MIN elevation, giving the elevation range of the outcrop, in metres.
MEAN	The mean elevation value of all pixels within or partially within the area of the outcrop, taken from the HRSC DTM, in metres.
Image_ID	ID of FRT CRISM data from which the observation is taken.
Type	Arbitrary geographical designation for the outcrop, used to approximately group for ease.
Band_1.4	Location of the 1.4 M-OH band
Band_1.9	Location of the 1.9 H2O band
Band_2.3	Location of the 2.3 M-OH band
Band_2.4	Location of the 2.4 M-OH band
CENTROID_X	Longitude of centre point.
CENTROID_Y	Latitude of centre point.

Mound_Clay_Detections_Points.zip

Point shapefiles showing the location and composition of bright-toned mound outcrops that contain phyllosilicate minerals observed using FRT CRISM hyperspectral data. This is identical to the previous dataset, but in point form.

Field Header	Description
OBJECTID	Numerical designation for each outcrop.
Shape	Points (across all individuals).
Target_ID	ID of FRT CRISM data from which observation is taken, followed by a numerical designation of an individual spectrum taken from the area of interest this shape covers.
MIN	The value of the pixel with minimum elevation within or partially within the area of the outcrop, taken from the HRSC DTM, in metres.
MAX	The value of the pixel with maximum elevation within or partially within the area of the outcrop, taken from the HRSC DTM, in metres.
RANGE	The MAX subtract MIN elevation, giving the elevation range of the outcrop, in metres.
MEAN	The mean elevation value of all pixels within or partially within the area of the outcrop, taken from the HRSC DTM, in metres.
Image_ID	ID of FRT CRISM data from which the observation is taken.
Type	Arbitrary geographical designation for the outcrop, used to approximately group for ease.
Band_1.4	Location of the 1.4 M-OH band
Band_1.9	Location of the 1.9 H2O band
Band_2.3	Location of the 2.3 M-OH band
Band_2.4	Location of the 2.4 M-OH band
CENTROID_X	Longitude of centre point.
CENTROID_Y	Latitude of centre point.

Stacked_Profiles_MP_Topography.csv

.csv file describing the topography within the stacked transect area.

Field Header	Description
Distance	Distance from the transect start, in kilometres.
Elevation	Elevation of the point
LINE_ID	ID of each transect (1-237)

Stacked_Profiles_MP_Outcrops.csv

.csv file describing the topography within the stacked transect area.

Field Header	Description
Type	Either Bright_Outcrop_Min (minimum elevation of a bright outcrop), Bright_Outcrop_Max (maximum elevation of a bright outcrop), Fe_Mg (detection of Fe/Mg-rich clays on the plateau), Al (detection of Al-rich clays on the plateau), or Mound_CRISM (detection of clays of any kind within a mound).
Distance	Distance from the transect start, in kilometres.
Elevation	Elevation of the point

Mound_Spectra.txt

Text file containing spectra for each outcrop on a mound that has a corresponding clay detection in CRISM; column headers correspond to Target_ID in Mound_Clay_Detections_Points.zip and Mound_Clay_Detections_Polygons.zip. Formatted as ASCII for easy ingestion into an ENVI spectral plot.
